# Maskless inverted pyramid texturization of silicon

**DOI:** 10.1038/srep10843

**Published:** 2015-06-02

**Authors:** Yan Wang, Lixia Yang, Yaoping Liu, Zengxia Mei, Wei Chen, Junqiang Li, Huili Liang, Andrej Kuznetsov, Du Xiaolong

**Affiliations:** 1 Key Laboratory for Renewable Energy, Beijing Key Laboratory for New Energy Materials and Devices, National Laboratory for Condensed Matter Physics, Institute of Physics, Chinese Academy of Sciences, Beijing, 100190, China; 2 Department of Physics, Centre for Materials Science and Nanotechnology, University of Oslo, PO Box 1048 Blindern, Oslo, NO-0316, Norway

## Abstract

We discovered a technical solution of such outstanding importance that it can trigger new approaches in silicon wet etching processing and, in particular, photovoltaic cell manufacturing. The so called inverted pyramid arrays, outperforming conventional pyramid textures and black silicon because of their superior light-trapping and structure characteristics, can currently only be achieved using more complex techniques involving lithography, laser processing, etc. Importantly, our data demonstrate a feasibility of inverted pyramidal texturization of silicon by maskless Cu-nanoparticles assisted etching in Cu(NO_3_)_2_ / HF / H_2_O_2_ / H_2_O solutions and as such may have significant impacts on communities of fellow researchers and industrialists.

Silicon texturization is an important topic of modern science and technology, particularly appealing in photovoltaics (PV)^1^,^2^,^3^. Random pyramid arrays with a reduced reflectivity of 10% can be readily obtained via anisotropic etching of (100)-oriented crystalline silicon (c-Si) in alkaline solutions^4^, which is widely used in c-Si PV cell manufacturing^5^,^6^,^7^.The black silicon (B-Si) with even lower reflectivity, down to 2%, is widely obtained by metal-assisted chemical etching^8^. However, B-Si is still not suitable for PV cells because of its high recombination rates due to the nanostructures^9^. The so-called inverted pyramid arrays, outperforming pyramid arrays and B-Si in PV because of their superior light-trapping and structure characteristics^10^,^11^,^12^, can currently only be achieved using more complex techniques involving lithography, laser processes, etc^13^,^14^,^15^,^16^,^17^,^18^. This complexity and corresponding extra costs hinder the implementation of inverted pyramidal structures in mass production. Here, we demonstrate the use of maskless Cu-nanoparticles (NPs) assisted anisotropic etching of c-Si in Cu(NO_3_)_2_/HF/H_2_O_2_/H_2_O, resulting in excellently performing inverted pyramidal arrays. Importantly, we do not only report a unique technical solution but uncover the underlying mechanisms, interdisciplinary in nature. In particular, due to a limited electron capturing ability of Cu^2+^ and a difference of electron supplying rates in Si (100) and (111) planes, Cu-NPs population as attached to c-Si, appear to be a function of the crystallographic plane orientation. Tuning the density of Cu-NPs on Si (100) and (111) planes naturally allows sui generis carrier transport balance enabling the anisotropic etching. Notably, our technique is compatible with a majority of PV production lines and as such may trigger a new era of using inverted pyramidal texturization of Si.

## Results and Discussion

Wafer-scale arrays of Si inverted pyramids were fabricated via a Cu-NPs-assisted anisotropic etching technique in a Cu(NO_3_)_2_/HF/H_2_O_2_/H_2_O mixture at 50 °C. The underlying principles are based on the electrochemical reaction between Si and Cu^2+^/Cu-NPs^19^,^20^. The driving force of this electrochemical reaction is the electrochemical potential difference between Si and Cu^2+^/Cu-NPs. The reaction can be described as two half-cell reactions just like the well-known metal-assisted chemical etching method for fabricating various Si nanostructures^21^,^22^,^23^,^24^,^25^.

Cathode reaction:


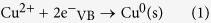



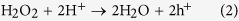


Anode reaction:


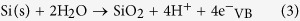



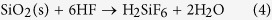


In this case, the Si/Cu(NO_3_)_2_/H_2_O_2_/HF system is composed of a corrosion-type redox couple: the cathodic reduction of Cu^2+^ ions with H_2_O_2_ as well as the anodic oxidation and dissolution of Si beneath the deposited Cu-NPs. Cu^2+^ ions capture electrons from the vicinity of the Si substrate, aggregate and form NPs. In this manner, Cu-NPs nucleated originally on the Si surface attract electrons from Si and become negatively charged because Cu is more electronegative than Si. These negatively charged Cu-NPs grow further by attracting the Cu^2+^ ions from the solution^19^,^20^,^26^. During this process, Si atoms underneath the Cu-NPs are continuously oxidized and etched by the HF. Simultaneously, the reduction of H_2_O_2_ on the Cu-NPs is indispensable to induce anisotropic Cu-NPs’ deposition onto the Si surface and guarantee the formation of inverted pyramid arrays, which will be interpreted in the following sections. The inverted pyramid arrays were cleaned using concentrated nitric acid in a sonication bath for at least 20 min to remove all of the residual Cu-NPs. After the nitric acid bath, no Cu-NPs were observed by scanning electron microscopy (SEM) analysis and no Cu peaks appeared in the energy-dispersive X-ray spectra of the inverted pyramid arrays.

**Figure 1:**
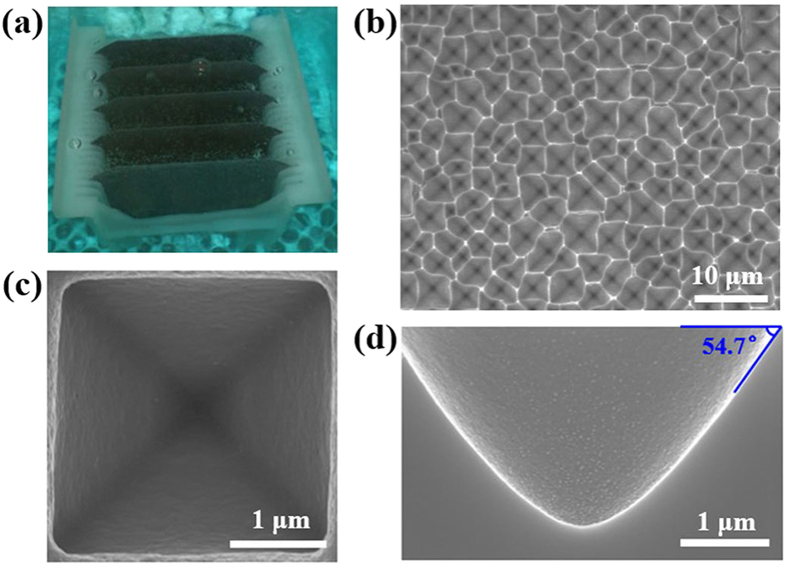
A photo of Cu-NPs-assisted etching process and SEM images. (**a**) 156 mm × 156 mm c-Si wafers being etched in a Cu(NO_3_)_2_/HF/H_2_O_2_/H_2_O etching bath. (**b**) SEM top-view image of the inverted pyramid arrays for 15 min processing. (**c**) Magnified SEM top-view image of an individual inverted pyramid processed for 15 min. (**d**) SEM cross-sectional view of an individual inverted pyramid processed for 15 min.

The inverted pyramid arrays fabricated using this approach were regular and consistent throughout the batches and across large areas, up to wafer-scale. Fig. 1(a) shows a photo of several 156 mm × 156 mm Si (100) wafers being etched in the Cu(NO_3_)_2_/HF/H_2_O_2_/H_2_O solution. The cross-sectional SEM image of the inverted pyramid shown in Fig. 1(d) reveals that the angle between the facet of the inverted pyramid and the (100) surface is 54.7°, indicating that the facets are terminated with Si (111) planes. As shown in Fig. 1(b), the inverted pyramid arrays are random because of the original irregular morphology and saw damage on Si; however, the surface is fully covered by random inverted pyramids and is regular when considered as a whole. The length of the inverted pyramids’ bottom side varies within 2–6 μm, and the etching depth is in the range of 1–5 μm. Fig. 1(c) shows a standard inverted pyramid with (111) sidewalls. The key parameters of the reaction were identified using p-type (100)-oriented, nominally 1-3 Ω cm c-Si as the etching wafer, but the process was successfully tested for n-type material too, naturally adjusting the solvent. In general, the morphology of the inverted pyramid arrays was well controlled by adjusting the etching time, the concentration of Cu(NO_3_)_2_/HF/H_2_O_2_ and the etching temperature.

**Figure 2:**
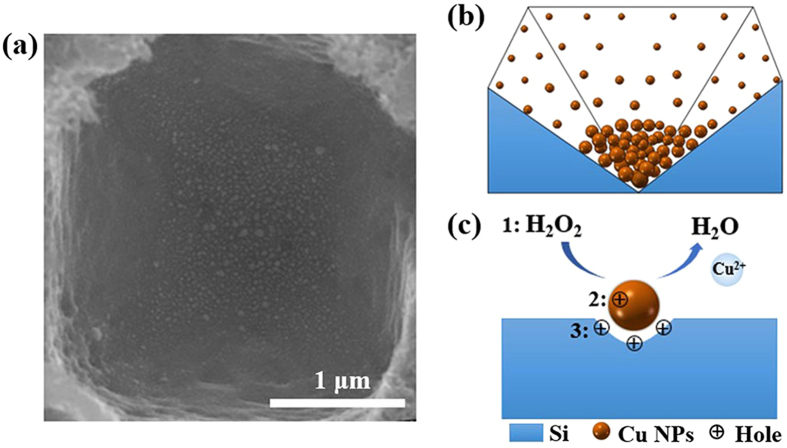
SEM image and schematics of Cu NPs assisted anisotropic etching. (**a**) SEM top-view image of an individual inverted pyramid via etching for 1 min. (**b**) Schematics of the anisotropic deposition of Cu NPs. (**c**) Schematics of the H_2_O_2_ reduction process and the mechanisms for the holes injection.

Up to date, the anisotropic wet chemical etching of c-Si has been done in alkaline solutions, resulting in random pyramid arrays, whereas isotropic wet chemical etching is commonly observed in HF based solutions^4^. In our technique, the anisotropic deposition of Cu-NPs from the Cu(NO_3_)_2_/HF/H_2_O_2_/H_2_O mixture is the key step in the synthesis of the inverted pyramid arrays without any mask. In Fig. 2(a), the early form of one inverted pyramid is demonstrated, where more and larger Cu-NPs are observed on Si (100) surface and fewer and smaller Cu-NPs are observed on Si (111) surfaces. This orientation-dependent population of Cu-NPs occurs due to a low reduction potential of Cu^2+^, exhibiting a limited ability to withdraw electrons directly from Si at room temperature^27^. Notably, rising temperature (to 50 °C) enhances the probability of the electron capturing by Cu^2+^ ions, leading to the nucleation of Cu-NPs on c-Si. Importantly, the electronic properties of (100) and (111) planes in c-Si are different in terms of the surface bond densities^4^,^28^,^29^. Accounting that, the number of electrons “available” on Si (100) planes is greater than that on the Si (111) planes, Cu-NPs preferentially nucleate on Si (100) plane because of the higher probability of electron capturing by Cu^2+^ ions. Further growth may be facilitated by the interaction between Cu^2+^ ions and these negatively charged Cu-NPs, resulting in faster growth of Cu-NPs on Si (100) plane than that on (111) planes. Fig. 2(b) is a simulated diagram that shows the pattern of anisotropic Cu-NPs’ deposition. As a result, Si atoms underneath the Cu-NPs are preferentially oxidized and then etched by HF, leading to a faster etching along Si <100> directions.

Notably, H_2_O_2_ plays an important and special role in the selective etching too, as previously mentioned. Energetically, the electrochemical potential of H_2_O_2_ is much more positive than either the valence band of Si or the reduction potential of Cu^2+^/Cu-NPs. Thus, the presence of H_2_O_2_ may promote holes injection into the valence band of Si as well as into the Cu-NPs. Although the Si etching by HF does occur when a Si substrate is subjected to an HF/H_2_O_2_ solution, the etching rate is less than 10 nm/h in an etchant with a high concentration of H_2_O_2_. In practice, the presence of a noble metal is necessary for accelerating Si etching in HF/H_2_O_2_ solutions^22^. In other words, the observed preferential reduction of H_2_O_2_ is attributed to the surface of the metal rather than to the surface of the bare Si^22^. Therefore, the reduction of H_2_O_2_ (labeled as “1” in Fig. 2(c)) in the Si/Cu(NO_3_)_2_/H_2_O_2_/HF system is likely to occur on the surface of the Cu-NPs too. When moderate amounts of H_2_O_2_ are introduced, Cu-NPs are deposited anisotropically, which assists in the etching of inverted pyramids by HF; thus, the injected holes are consumed via two pathways. The holes are consumed by the Cu-NPs, ensuring kinetic equilibrium between the deposition and dissolution of Cu NPs (as labeled by “2” in Fig. 2c). Some holes also diffuse through the Cu-NPs and are injected into the Si substrate (labeled by “3” in Fig. 2c) selectively accelerating the etching of c-Si beneath the Cu-NPs.

In the absence of H_2_O_2_, the reduction of Cu^2+^ will be faster, allowing more Cu-NPs to be formed at the Si surface because of unlimited supply of Cu^2+^ ions. The Cu-NPs quickly grow larger and cover the majority of the Si wafer surface^30^. This situation induces overlapping regions of oxidation and, accordingly, faster isotropic Si etching. A thin Cu film will be formed very quickly at 50 °C; this film will inhibit the etching of Si by acting as a barrier between the Si and the etchants. At high concentrations of H_2_O_2_, the oxidation of Cu-NPs is faster than the reduction of Cu^2+^, resulting in the deposition of a few small Cu-NPs randomly on the Si surface; therefore, the original morphology of the Si does not substantially change, except for the formation of a few shallow nano-pits. On the basis of the interaction between H_2_O_2_ and Cu-NPs and their roles in the process, inverted pyramid arrays were obtained for the first time using a simple and low-cost maskless chemical etching.

**Figure 3:**
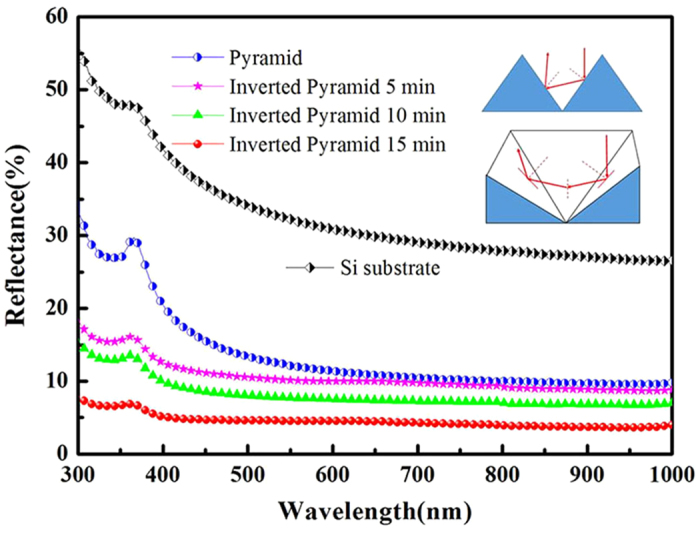
Reflectance spectra of pyramid arrays and the inverted pyramid arrays obtained via Cu NPs assisted anisotropic etching for 5 min, 10 min and 15 min. The inset shows schematics of the light path in the normal pyramidal structure and an individual inverted pyramid before being reflected away.

After the etching process, the hemispheric total reflectance for normal incidence was measured using a Varian Cary 5000 spectrophotometer with an integrating sphere. The reflectance spectra of the inverted pyramid arrays prepared using different etching times are shown in Fig. 3, illustrating the tunability of our technique. Notably, our texturization reduced the mean reflectivity of c-Si (over the wavelength range from 300 to 1000 nm) down to ~4.4% without any antireflective coating as compared to much higher reflectivity from the normal pyramid arrays (see Fig. 3). The short wavelength spectral response analysis indicates that approximately 37% of the incoming light undergoes a triple bounce due to its interaction with the adjoining planes of the inverted pyramid geometry before being reflected away^11^. As shown in the inset in Fig. 3, the incident light, which strikes one face of the inverted pyramid, is reflected (the first bounce) onto an orthogonal plane, which is then reflected (the second bounce) onto the face complementary to the original point of impact and finally reflected away (the third bounce), while a double bounce of most incident light was observed in the pyramid geomtry^11^. The triple bounce increases the path length of the light, which induces an increased absorption of the incident light. The reduced front surface reflectance, together with the increased light absorption, makes inverted pyramid arrays an attractive and efficient light-trapping geometry. Moreover, the recessed structure is more suitable for covering and filling, such as for the coverage of amorphous Si in heterojunction solar cells and the filling of metal electrodes in photovoltaic devices.

In summary, we discovered a method of maskless texturization of c-Si with so-called inverted pyramid arrays that can be immediately uptaken for industrialization. In comparison with the pyramidal structure, which is widely used for Si solar cells, the inverted pyramidal structure has the same micrometer scale but lower reflectivity and higher light absorption, along with a shorter etching time and a lower etching temperature during the texturing process; compared with the B-Si obtained by the noble metal assisted chemical etching, inverted pyramidal structures are achieved by the cheaper metal, Cu-NPs assisted chemical etching. Moreover, inverted pyramid not only has superior structural characteristic but also ensures low recombination rates. Furthermore, the generality and scalability of our technique are very promising for the fabrication of other advanced Si-based devices. Last but not least, the underlying principles governing the etching process are understood. In particular, the preferential etching along <100> directions is realized by a charge transfer assisted with Cu-NPs.

## Methods

Inverted pyramid arrays were originally etched from commercial 156 mm × 156 mm, boron-doped (1-3 Ω cm), (100)-oriented c-Si wafers via Cu NPs assisted anisotropic etching. Before being etched in the solution, the wafers were thoroughly rinsed in acetone to remove any organic contaminants and then rinsed with deionized water. The inverted pyramid arrays were obtained when the wafers were etched for 15 min at 50 °C in a polytetrafluoroethylene container filled with 5 mM Cu(NO_3_)_2_, 4.6 M HF and 0.55 M H_2_O_2_. Residual Cu-NPs were removed using concentrated nitric acid in a sonication bath for at least 20 min. The Si wafers were thoroughly rinsed with deionized water and dried under flowing nitrogen. The morphologies and structures of the wafers were characterized using a Hitachi S-4800 scanning electron microscope. The hemispheric total reflectance for normal incidence was measured using a Varian Cary 5000 spectrophotometer with an integrating sphere.

## Additional Information

**How to cite this article**: Wang, Y. *et al.* Maskless inverted pyramid texturization of silicon. *Sci. Rep.* **5**, 10843; doi: 10.1038/srep10843 (2015).
